# Clinical utility of the peritoneal pathologic regression in gastric cancer patients associated to peritoneal metastasis. a study protocol

**DOI:** 10.1515/pp-2025-0005

**Published:** 2025-09-08

**Authors:** Silvia Guerrero-Macías, María Eugenia Manrique-Acevedo, Carlos E. Bonilla, Magda Vargas Diaz, Xavier Delgadillo

**Affiliations:** Peritoneal Malignancies Unit, Cancer Treatment & Research Center (CTIC), Bogota, Colombia; Gastrointestinal & Neuroendocrine Tumors Unit, Cancer Treatment & Research Center (CTIC), Bogota, Colombia; Pathology Oncology, Cancer Treatment and Research Center (CTIC), Bogota, Colombia; Unité Spécialisée de Chirurgie, Centre Médico Chirurgical Volta, La Chaux-de-Fonds, Switzerland

**Keywords:** pathologic regression grading score, gastric cancer, peritoneal metastasis

## Abstract

**Objectives:**

Peritoneal regression grade score (PRGS) has emerged a scoring system designed to measure the extent of residual disease following systemic or intraperitoneal therapies in patients with carcinomatosis. Higher (3–4) PRG-Scores match with a mediocre treatment response and prognosis. Conversely, lower grades (1–2) response are linked to significantly longer overall and progression-free survival periods. This study explores the utility of PRGS in assessing prognosis and optimizing therapeutic strategies for patients with peritoneal metastasis secondary to gastric malignancy.

**Methods:**

This is a prospective cohort study, including patients with gastric cancer and peritoneal metastasis undergoing chemotherapy with intent for subsequent cytoreductive surgery. The primary endpoint of the study is to assess the pathological response of peritoneal involvement to primary chemotherapy according to the PRGS. Secondary objectives are to correlate PRGS with some clinical, pathological and molecular features (MMR, PDL1, CPS, HER2) as well as with other clinical and biochemical markers related to chemotherapy response.

**Results:**

This protocol summarizes the current scientific evidence regarding the effectiveness of the PRGS in assessing peritoneal response to targeted therapies. It further hypothesizes its potential utility in evaluating the effects of systemic therapies for gastric cancer with peritoneal metastases, while also defining inclusion and exclusion criteria and outlining a flowchart for its implementation.

**Conclusions:**

Our final endpoint is to expand PRGS applications to curative settings and identify factors such as tumor biology and chemotherapy regimens that may guide patient selection for adjuvant hyperthermic intraperitoneal chemotherapy (HIPEC) in gastric cancer with peritoneal metastasis.

## Introduction

For decades, systemic chemotherapy has been the cornerstone treatment for gastric cancer with peritoneal metastases.

Recently, alternative strategies like cytoreductive surgery (CRS) combined with hyperthermic intraperitoneal chemotherapy (HIPEC) have been explored in select cases to reduce tumor burden and enhance exposure of peritoneal metastases to cytotoxic drugs [1].

Recent up-dating in multicenter studies, a large retrospective series and some clinical trials had evaluated the benefit of CRS associated to HIPEC in patients with GC and peer PM (peritoneal metastases) [2].

Preoperative imaging with computed tomography scanner (CT-Scan) or magnetic resonance imaging (MRI) of the abdomen and pelvis is mandatory for staging this category of patients; images provide useful information for grading but do not accurately predict the rate of peritoneal metastasis.

Various publications report the identification of peritoneal involvement not documented by imaging in a range of 18–41 % [3], 4], which is more relevant in countries such as ours with a high incidence of patients in advanced stages and a higher probability of peritoneal involvement.

Staging laparoscopy has positioned itself as a useful tool to complete the staging process prior to initiating chemotherapy with curative or conversion intent in patients with gastric cancer and peritoneal involvement [5].

This procedure not only enables the feasibility of adequate cytoreduction, but also allows biopsies of suspicious lesions for histopathological confirmation and comparison with samples taken after chemotherapy to calculate the peritoneal regression applying a very useful grading score (PRGS).

PRGS has so far been used in the palliative setting of peritoneal metastasis of gastric origin, to assess the pathological response secondary to peritoneal targeted therapies, like pressurized intra-peritoneal aerosol chemotherapy (PIPAC), but assessing the peritoneal response to intra venous chemotherapy in a therapeutic setting, will permit to make a correlation with some of the variables that influence the peritoneal response rate to the systemic therapy.

Factors identification, could assist in the selection of systemic or a very specific targeted therapy, as well as the possibility of applying PIPAC in other patients with similar clinical or pathological characteristics.

Additionally, the correlation of this response with oncological outcomes will permit us to add tools to determine the prognosis of patients with gastric cancer associated to peritoneal metastasis.

## Methods

We propose two aim-pathways to assess, correlate and describe our objectives:

### Primary Objective(s)

– To assess the pathological response of peritoneal involvement to primary chemotherapy based on PRGS in patients with gastric cancer undergoing staging laparoscopy for potentially cytoreductive surgery.

### Secondary Objective(s)

– Correlate PRGS with some clinical, pathological and molecular characteristics (MMR, PDL1, CPS, HER2) in patients with gastric cancer.
– Correlate PRGS with other clinical, radiological and biochemical markers resulting on response to systemic chemotherapy.
– Describe the PRGS according to different chemotherapy protocols and target therapies administrated to patients with gastric cancer.
– Describe some oncological outcomes according to the degree of peritoneal pathological response (PRGS).

## Study design

**Figure 1:**
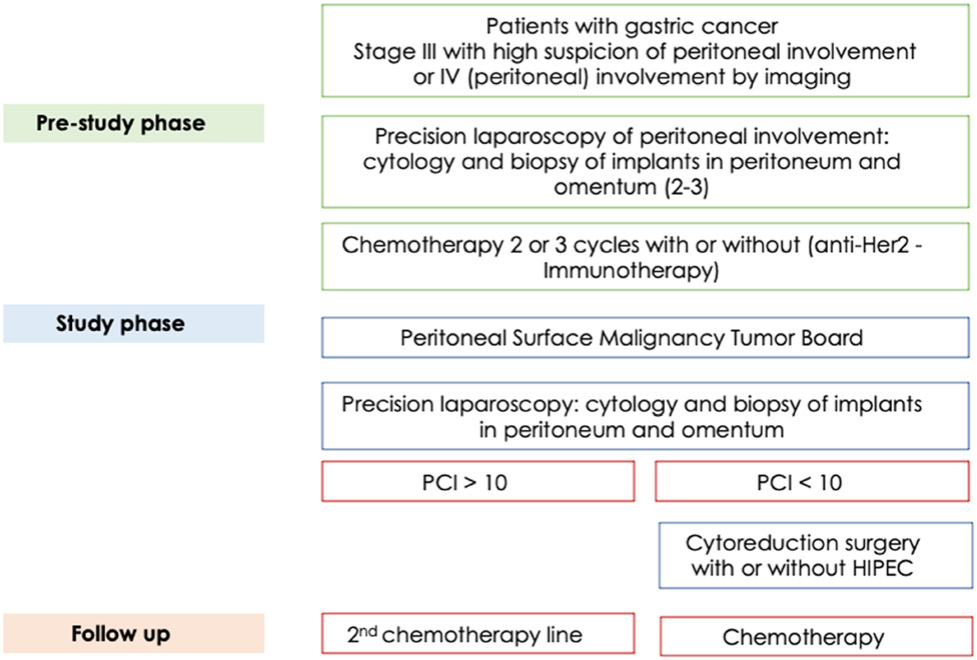
Study organization phases and flow chart.

The layout matching with our study design flow-chart and description are represented in Figure 1.

### Inclusion criteria

– Precision surgical laparoscopy to document the peritoneal involvement before the beginning of chemotherapy.
– Completion of the proposed chemotherapy protocol.
– Precision surgical laparoscopy after chemotherapy.
– Peritoneal cytology and samples biopsies of peritoneal implants obtained during laparoscopies to assess the peritoneal regression grade score.

### Exclusion criteria

– Synchronous neoplasia
– Extraperitoneal metastasis
– Pregnancy

### Perioperative chemotherapy

Neoadjuvant treatment consisting of three months intravenous chemotherapy, patients received oxaliplatin, capecitabine (XELOX) or oxaliplatin, fluorouracil/leucovorin (FOLFOX-4) or fluorouracil, leucovorin, oxaliplatin and docetaxel (FLOT).

Administration of immunotherapy drugs or anti-HER2 agents was submitted to the molecular profile of the patients, which has been given early in the staging process.

### Procedure

Surgical precision laparoscopy after intravenous chemotherapy has been performed between two to three weeks following the chemotherapy’s protocol completion.

Peritoneal cytology samples obtained at least from two different quadrants as mandatory surgical maneuver. In case of absence of ascites or lack of peritoneal free fluids, 1 L of a 0.9 % saline solution irrigation may be used to facilitate cytological sample collection.

Peritoneal biopsies should be obtained from four different regions of the peritoneal cavity, including at least three bite-samples taken from a 4 cm^2^ of the chosen sectors to be biopsied.

**Figure 2:**
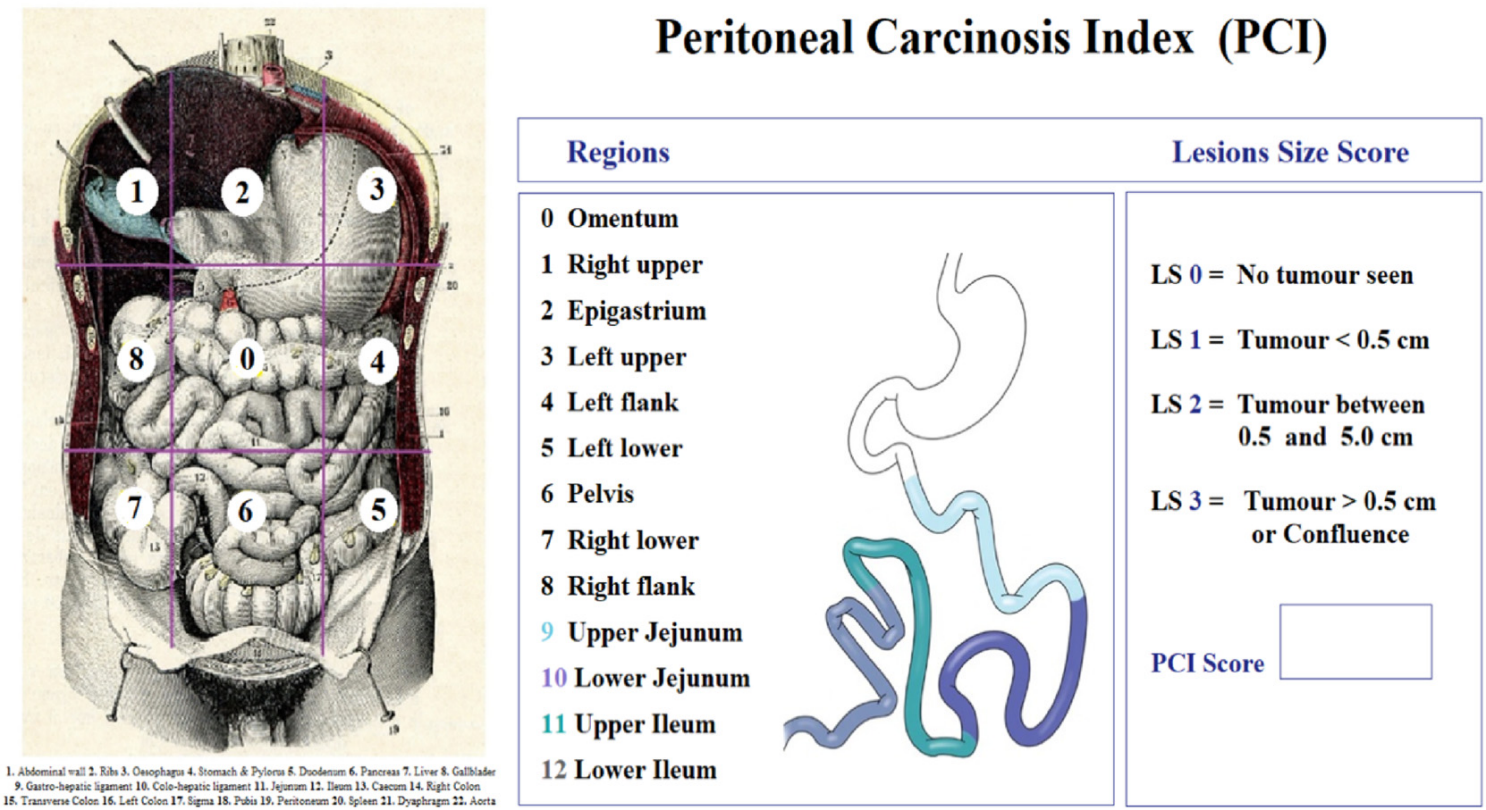
Peritoneal carcinosis evaluated by PCI including Z=0 (omentum) before biopsies. (image provided courtesy *X. Delgadillo* [10] *& adapted from Sugarbaker P.*[5]).

It is mandatory to include the Zone=0 (Omental zone) as described on the international peritoneal parcinosis index [6] as shown in Figure 2.

Non-energy cutting instruments should be used to minimize sample damage and ensure the integrity of the tissue for histopathological analysis.

### Sample size

The sample size required for this study was calculated assuming an event prevalence of 15 % (p=0.15), with a confidence level of 95 % (Z=1.96) and a margin of error of 5 % (d=0.05). An initial sample size of 196 patients was obtained.

### Sample analysis

**Figure 3:**
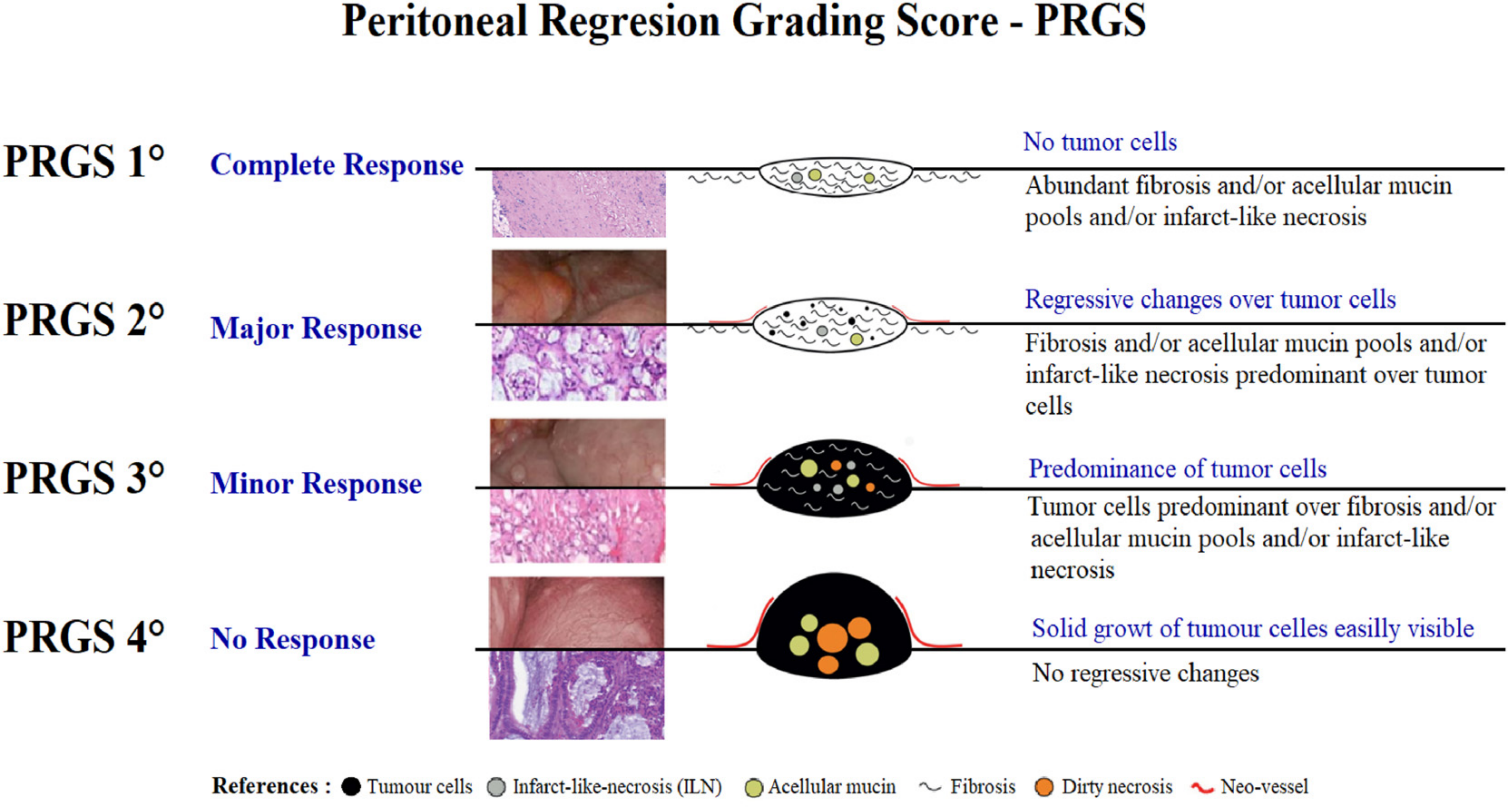
PRGS - peritoneal regresion grading score, adapted from Solaß W. et al. and modified by X. Delgadillo et al.[10].

The PRGS categorizes treatment response into four grades [7], [8], [9], [10] as shown in Figure 3.

– Grade 1: Complete response with no macroscopic disease. This optimal response is associated with the highest likelihood of long-term disease-free survival.
– Grade 2: Major response with evident regressive features and minimal residual tumor cells.
– Grade 3: Minor response with some regressive features but predominantly residual tumor cells.
– Grade 4: No response, with tumor cells showing no regressive features. This indicates a poor prognosis and higher likelihood of disease progression.

## Strengths and limitations of this study

### Strengths

– Comprehensive Pathological Evaluation: Enables assessment of the peritoneal pathological response in the context of various clinical and oncological features.
– Informed Decision-Making: Supports tumor board discussions for personalized treatment planning in gastric cancer patients with peritoneal involvement.
– Optimized Treatment Flow: Ensures there is no delay between primary chemotherapy and subsequent oncological treatments.

### Limitations

– Diverse Chemotherapy Protocols: Variability in perioperative systemic chemotherapy regimens may influence outcomes.
– Standardization Challenges: Lack of uniformity in the precision laparoscopy protocol, including the number of cytologies and biopsies collected.
– Observer Variability: Potential differences in inter-observer interpretation of peritoneal involvement and peritoneal cancer index (PCI) calculations.
– Operative Reporting: Inconsistent documentation in surgical reports may limit the accuracy of data analysis.

## Statistical analysis

Data will be obtained from medical records and pathology reports and entered into a study-specific database using the Research Electronic Data Capture (REDCap^®^) platform.

Descriptive Statistics concerns continuous variables that will be summarized as medians and interquartile ranges (IQR), with the 25th and 75th percentiles reported.

In case of missing data more than 5 %, the strategy of multiple imputations will be used to reduce the loss of patients for the statistical analysis.

## Survival analysis

– Median overall survival will be calculated using incidence density rates, where the numerator is the number of deaths from any cause, and the denominator is the total patient follow-up time.
– Survival functions will be estimated and graphically represented using the Kaplan–Meier method, with 95 % confidence intervals.

## Association analysis

– Cox proportional hazards models will be used to evaluate associations between oncological treatment protocols and outcomes.
– Covariates with a preliminary bivariate association (p-value < 0.20) will be included in the multivariate model.
– Additional covariates identified in the literature as potential prognostic factors will also be incorporated.

## Software

– All analyses will be conducted using R - Project software (version 3.6.2).

## Monitoring the study

During the trial, a representative of the Research Unit will maintain regular communication with the principal investigator, to provide information and support, confirming that the investigational team is adhering to the protocol, that data are being accurately and timely recorded in the system; to verify source data (comparison of the data in the Redcap with the patient’s medical records) including verification of informed consent.

## Ethics approval

The study complies with the following ethical standards:

– Good Clinical Practice Guidelines from the International Committee on Harmonisation.
– The Declaration of Helsinki for ethical principles in medical research.
– Guidelines from the Council for International Organizations of Medical Sciences (CIOMS).
– Resolution 008430 of 4 October 1993 by the Ministry of Health of Colombia.

The Research Ethics Committee of the CTIC approved this study protocol under Resolution CEI-00786-21, in accordance with Colombian Law 0014–21. The committee determined that the research involves no risk and thus does not require informed consent.

Conflicts of interest: All authors declared that they had no conflicts of interest.

Sources of funding: this work was self-funded by the authors.

Patient and public involvement: Patients and the public were not involved in the design, conduct, reporting, or dissemination plans of this research.

## Discussion

Diagnostic surgical laparoscopy in the staging process for gastric cancer is useful to accurately determine peritoneal involvement and define the intention of oncological treatment [3].

The efficacy of this procedure for the detection of carcinomatosis ranges of 95–100 % [5], 11] and it is estimated to allow re-staging and reduces the number of non-therapeutic laparotomies in about 44 % [12].

However, the demonstrable benefit of neoadjuvant therapy for patients with resectable advanced gastric cancer [13], and the possibility of providing surgical management in the setting of limited metastatic disease, expanded the indications for laparoscopy.

Laparoscopic evaluation provides critical information when imaging studies such as CT or MRI yield inconclusive results [12].

Pathology samples obtained during the initial laparoscopy are instrumental in calculating the PRGS by comparing pre- and post-chemotherapy pathology findings [14]. This regression refers to the reduction in size or disappearance of tumor components in the peritoneum in response to systemic treatment.

The PRGS provides an objective measure of the amount of residual disease remaining after systemic or direct therapies to the peritoneum.

Higher grades (3–4) indicate worse response to treatment and worse prognosis, with earlier recurrence.

Grade 1 and 2 responses are associated with significantly longer overall and progression-free survival [7], 14]. However, the size of residual disease deposits remains the strongest prognostic indicator.

Emerging evidence aims to extend the use of PRGS and incorporate additional factors such as tumor biology and chemotherapy protocols. It has been questioned whether PRGS results, combined with other markers of chemotherapy response, could guide therapeutic decisions in the curative setting, expanding its utility beyond palliative care.

## Conclusions

Our research study pursues to validate and expand the use of PRGS in cases of curative setting, focusing on its role in predicting treatment response and prognosis in gastric cancer patients with peritoneal metastasis.

By incorporating tumor biology, chemotherapy protocols, and other associated factors, this study aims to refine patient selection criteria for adjuvant HIPEC. These findings could enhance personalized treatment planning and improve oncological outcomes in this challenging patient population**.**

Finally, PIPAC delivery of drugs remains our proposal in case of palliative situation of gastric cancer peritoneal metastasis.
